# Synchronization of Neurophysiological and Biomechanical Data in a Real-Time Virtual Gait Analysis System (GRAIL): A Proof-of-Principle Study

**DOI:** 10.3390/s24123779

**Published:** 2024-06-11

**Authors:** Stefan A. Maas, Tim Göcking, Robert Stojan, Claudia Voelcker-Rehage, Dieter F. Kutz

**Affiliations:** Department of Neuromotor Behavior and Exercise, University of Münster, 48149 Münster, Germany; stefan.maas@uni-muenster.de (S.A.M.); tim.goecking@uni-muenster.de (T.G.); robert.stojan@uni-muenster.de (R.S.); claudia.voelcker-rehage@uni-muenster.de (C.V.-R.)

**Keywords:** ecological validity, instrumented treadmill, neuroimaging, neurorehabilitation, neuroergonomics

## Abstract

The investigation of gait and its neuronal correlates under more ecologically valid conditions as well as real-time feedback visualization is becoming increasingly important in neuro-motor rehabilitation research. The Gait Real-time Analysis Interactive Lab (GRAIL) offers advanced opportunities for gait and gait-related research by creating more naturalistic yet controlled environments through immersive virtual reality. Investigating the neuronal aspects of gait requires parallel recording of brain activity, such as through mobile electroencephalography (EEG) and/or mobile functional near-infrared spectroscopy (fNIRS), which must be synchronized with the kinetic and /or kinematic data recorded while walking. This proof-of-concept study outlines the required setup by use of the lab streaming layer (LSL) ecosystem for real-time, simultaneous data collection of two independently operating multi-channel EEG and fNIRS measurement devices and gait kinetics. In this context, a customized approach using a photodiode to synchronize the systems is described. This study demonstrates the achievable temporal accuracy of synchronous data acquisition of neurophysiological and kinematic and kinetic data collection in the GRAIL. By using event-related cerebral hemodynamic activity and visually evoked potentials during a start-to-go task and a checkerboard test, we were able to confirm that our measurement system can replicate known physiological phenomena with latencies in the millisecond range and relate neurophysiological and kinetic data to each other with sufficient accuracy.

## 1. Introduction

Gait is a key indicator of functional independence [1] and central for many activities of daily living [2]. Voluntary movements, such as walking, are induced by the smooth and complex interaction of the sensorimotor systems. These include, for example, the cerebellum adapting locomotion to perturbations by comparing planned with ongoing movements within the step cycle (e.g., [3]), the use of visual feedback during the transition from standing to walking and while walking (e.g., [4]), and the maintenance of a stable muscle tone, which is accomplished by receiving excitatory input from the cortex and inhibitory input from the basal ganglia (e.g., [5]). The harmonious interplay of neural mechanisms can be disrupted, resulting in gait disorders, which may be caused by diseases or aging (e.g., [6]). While there is generally a good understanding of gait, there are gaps in the understanding of certain gait abnormalities; for example, freezing of gait (FOG) in Parkinson’s disease (PD) [7] or age-related decline.

When addressing these gaps in gait research, it is crucial to consider the ecological validity of the experiments [8], as motor performance can differ between laboratory and everyday life [9]. This could entail transferring experimental results from controlled laboratories to less controlled environments [10], as walking usually takes place in an uncontrolled environment. A potential compromise between laboratory settings and real-world scenarios is the use of virtual reality (VR) laboratories. VR can provide complex environments that mimic real-world situations while minimizing risks compared to real-life walking situations [11,12,13]. As such, the Gait Real-time Analysis Interactive Lab (GRAIL; Motekforce Link, Amsterdam, The Netherlands) offers the opportunity for gait research using VR. The GRAIL makes it possible to create naturalistic and controlled environments through immersive VR in combination with a fully instrumented treadmill to facilitate gait research, and therewith advance earlier experimental setups [12,13].

While not a standard feature, the GRAIL presents a platform that might also allow for concurrent recording of brain activity during walking in healthy and patient populations during an ambulatory assessment. The measurement of brain activity during walking through mobile electroencephalography (EEG) [14] or mobile functional near-infrared spectroscopy (fNIRS) [15] synchronized with kinematic or kinetic gait measurements enables assessments of neuronal correlates in real-time. These multimodal assessments may facilitate a deeper understanding of gait disorders [6]. To ensure the accuracy of these insights, it is crucial to establish a real-time, highly synchronized integration of EEG, fNIRS with kinetic and kinematic data collection systems. Temporal synchronization is necessary as it is possible to observe evoked cerebral potentials and muscular reactions to standing or walking within milliseconds. A first muscular reaction to a perturbation, for example, can be measured after approximately 40 ms, while a cerebral evoked potential can be observed between 42 ms (standing) and 83 ms (walking) after a perturbation [16]. In order to accurately measure these reactions, it is essential to set up a synchronous measurement system that integrates all devices in real-time.

The primary objective of this study was to develop and validate a setup that allows for simultaneous collection and real-time synchronization of brain activity (via mobile EEG and fNIRS), kinetic, and kinematic gait measurements. This kind of setup will enable us to conduct fundamental research on brain correlates during online gait. Therefore, we first aim to confirm synchronization within the gait measurements such as force plates, motion capture system, and electromyography (EMG) and secondly a synchronization between the neurophysiological systems EEG and fNIRS and the gait measurements.

## 2. Materials and Methods

### 2.1. Reasons for the Design of the Measurement System

As previously stated, the GRAIL environment was selected as it offers unique capabilities for investigating gait in VR and the neurophysiological correlates of gait and gait-related disorders such as freezing of gait (FOG) in Parkinson’s disease (PD) or age-related changes, in a safe and controlled experimental environment. The GRAIL is equipped with force plates to monitor gait kinetics and a VICON movement analysis system to assess gait kinematics. In our setup, brain activity is recorded concurrently using both an EEG and fNIRS system to facilitate a deeper understanding of the neurophysiology of gait and gait disorders during online gait (for details see Section 2.2. Measurement Systems and Synchronization—The Components of the System).

**Figure 1:**
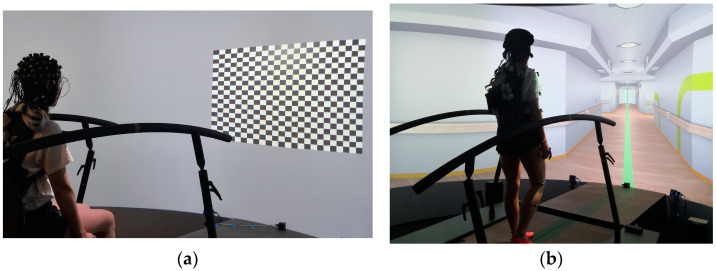
Virtual environments within the GRAIL. (**a**) Checkerboard setup used for the Checkerboard experiment. (**b**) Virtual hospital environment used for the Start-to-Go experiment.

When recording gait and neurophysiological data in real-time, two main problems can occur. First, delays within a system, and second, temporal delays between the different systems. The GRAIL system, depicted in Figure 1, comprises various components that can introduce delays within the system. Of particular relevance is the delay between the computational processes of the experimental computer and the display of visuals on the screen. This phenomenon can be attributed to both the processing of the image and its display onto the screen by the projectors. To address this problem, a photodiode is utilized in our system to detect signals that mark relevant changes in the visual environment. With respect to the second point, synchronization of the different systems, lab streaming layer (LSL) and a custom-programmed Raspberry Pi is used to transform the signals into triggers that are communicated to the EEG and fNIRS systems (for more details see Section 2.2. Measurement Systems and Synchronization—The Components of the System). The synchronization of events that are registered within the EEG, fNIRS, and motion capture system enable the analysis of related events from different systems to each other.

### 2.2. Measurement Systems and Synchronization—The Components of the System

GRAIL. The experiments were conducted within the confines of the GRAIL. The GRAIL system comprised a 3D instrumented split-belt treadmill (Motek Medical; Utrecht, The Netherlands) with a usable running surface measuring 1.0 m × 2.0 m (width × length). The setup comprised a semi-cylindrical 180° projection screen (Motek Medical; Utrecht, The Netherlands), measuring 2.4 m × 5 m (height × width). Virtual scenarios were displayed on the screen using three RGB projectors connected in series. An additional RGB projector directed images onto the treadmill’s surface. The GRAIL system integrates a number of components to capture motion data, including a marker-based, passive optical motion detection system (VICON Motion Systems Ltd.; Oxford, UK), two ground reaction force plates (Motek Medical; Utrecht, The Netherlands), and an external EMG measuring system (Cometa; Bareggio, Italy). The optical motion detection system features 10 infrared cameras (VICON VERO; VICON Motion Systems Ltd.; Oxford, UK), capturing videos at a frequency of 100 Hz. The cameras utilize retroreflective markers to track the movements of participants. For the purpose of this experiment, 39 retroreflective markers were placed in accordance with the VICON Plug-in Gait full body marker set [17]. Two independent force plates are positioned adjacent to each other in a mediolateral arrangement. These force plates captured 3D ground reaction forces at 1000 Hz and allow for the separate recording of each foot. In this study, we analyzed the force plate data, utilizing the higher recording frequency of the kinetic measurement system in contrast to the kinematic measurement system. The EMG measuring system comprises up to 16 wireless, miniature EMG amplifiers and a receiver, which is connected to the VICON measurement computer. For the purpose of this experiment, one amplifier was utilized, capturing data at a frequency of 1000 Hz. The amplifier was placed on the right musculus tibialis anterior in accordance to the SENIAM recommendations [18]. Additionally, a photodiode was integrated into the GRAIL (See: photodiode).

Virtual Scenarios. The virtual environments were developed using D-Flow software Version 3.36.2 (Motekforce Link, Amsterdam, The Netherlands), which allows the development of different virtual scenarios. In this study, we used a checkerboard and a hospital-like environment. The checkerboard scenario was used for experiment one “Checkerboard” (see Section 2.4.1. Task and Experimental Setup) and consisted of a fixation point (1.4 cm × 1.4 cm, corresponding to 0.32° × 0.32° of visual angle, colored grey) located at the center of a checkerboard pattern in the middle of the screen. The checkerboard consisted of 24 × 20 rectangles, each measuring 6 cm in width and 4.3 cm in height. Each checkerboard tile created a visual field of 1.43° × 0.96° with the chosen distance of 2.5 m to the projection screen. The center of the checkerboard was projected on the screen in front of the participant at a height of 1.25 m (approximately at eye level for a seated individual; Figure 1a). In order to provide a stimulus to the participants, the colors of each tile of the checkerboard underwent a change from white to black and vice versa. A virtual hospital corridor (Figure 1b) was used during the Start-to-Go experiment (See Section 2.4.1. Task and Experimental Setup) to simulate a more naturalistic environment during task performance.

**Figure 2:**
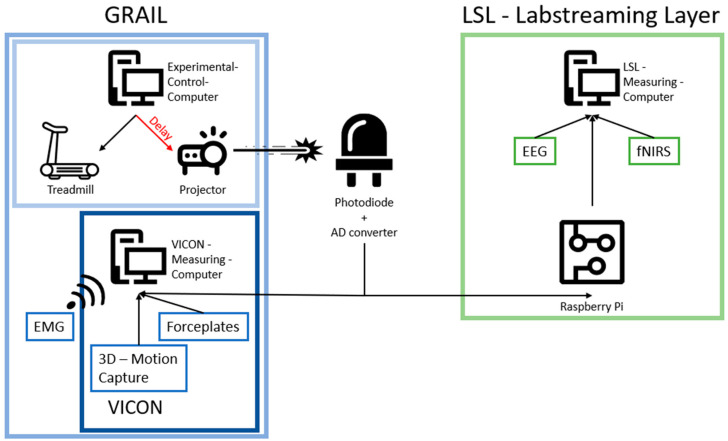
This illustration depicts the system integration within the experimental setup. Due to an unknown delay between the command from the experimental control computer and the actual projection on the screen, it is necessary to correct this in order to obtain accurate measurements in relation to the projection screen. This compensation is achieved by projecting a signal onto a photodiode, whose output is then transferred to the two measurement systems, namely the VICON environment and the lab streaming Layer.

Photodiode. In order to compensate for the delay between the signal from the experimental control computer and the actual projection on the screen, a photodiode was fixed to the bottom center of the projection screen of the GRAIL. This photodiode was used to receive a signal, which is sent by the experimental control computer via the projectors of the GRAIL (See Figure 2). Such a signal was sent regularly during the experiment to mark specific timepoints. By measuring the moment at which the signal appears on the photodiode, the delay of the projection could be compensated, since the signal underwent the same delay as the entire projection. These marked timepoints within the experiment could then be used to synchronize the different measurement systems. The placement of the photodiode at the bottom of the screen ensured that the signal was nearly imperceptible to participants. The signal was a binary signal, consisting of a white blinking dot. Each time a timepoint required marking, a byte of information was transmitted. This allowed for the use of different signals for different events; for example, a start signal at the beginning of each trial and a stop signal at the end of each. The photodiode signal was converted into a digital signal by an analogue–digital converter (Schmitt trigger) with adjustable properties, and then transmitted to the various measurement systems. The digital photodiode signal was recorded at a frequency of 1000 Hz via an analogue input channel in VICON and processed by a Raspberry Pi for the neurophysiological measurement systems (see LSL).

Nexus Software. All movement, EMG, the force plate data, and the additional photodiode signal were recorded simultaneously using a single measuring computer with VICON Nexus 2.12 software (VICON; Oxford, UK). The software exported various data types, including ground reaction force data, marker trajectories, joint angles, the amplified EMG signal, and the digital photodiode signal. The marker trajectories were provided in XYZ coordinates, with the origin at the center of the treadmill. The force data detailed the location and force vector for each individual force plate, in addition to a combined value. The EMG signals and the photodiode signal were output as a timeseries, showing activation values and the digital signal, respectively.

**Figure 3:**
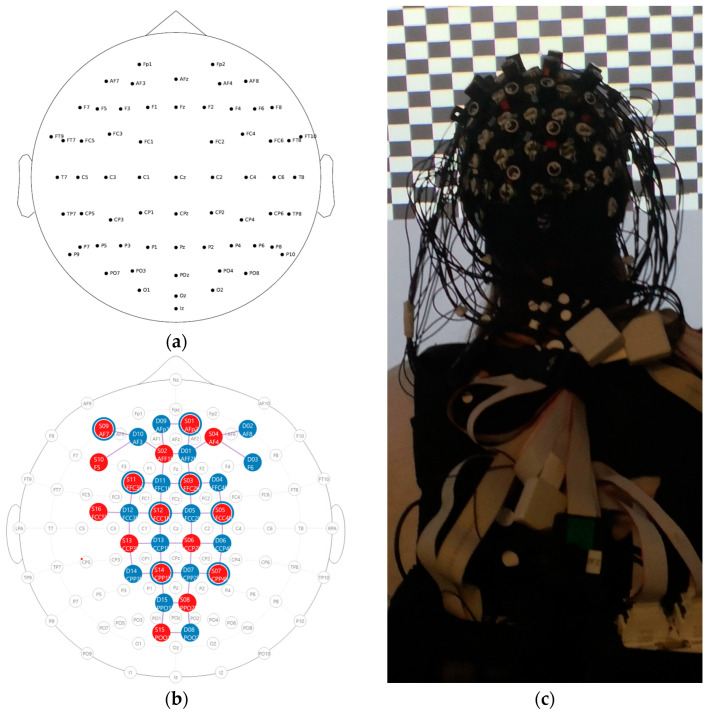
(**a**) EEG setup with 64 channels; (**b**) fNIRS setup with 16 emitters and 23 detectors (15 long, 8 short), resulting in 43 long channels and 8 short channels; detector D16 (right hemisphere) was used to implement 8 short channel detectors. (**c**) photograph of the EEG and fNIRS setup with the opaque cover removed.

EEG. EEG was recorded using a mobile amplifier, LiveAmp (Brain Products GmbH, Gilchingen, Germany), with 64 active electrodes arranged according to the 10–10 system [19] (Figure 3a). The FPz electrode served as a ground electrode, while the FCz electrode was used as an online reference. Data were recorded at a sampling rate of 500 Hz. Standard measures to ensure the data quality, such as an impedance check (<25 kOhm), were taken.

fNIRS. Two 8 × 8 NIRSport 2.0 systems (NIRx Medical Technologies, Glen Head, NY, USA) were employed in tandem for simultaneous recording of hemodynamic brain activity (Aurora Software Version 2023.9.3, NIRx Medical Technologies, Glen Head, NY, USA) during task performance. A total of 32 dual-tip optodes, including 16 light emitters and 16 detectors, were arranged in a bihemispheric configuration. The configuration spanned frontal, central, parietal, and occipital regions, adapted to the 10–5 electrode system and 64 electrode setup described above [20] (Figure 3b). Sources continuously emitted infrared light at two wavelengths (760 nm and 850 nm) with a modulation frequency of 5.086 Hz. Source-detector inter-optode distance was approximately 3–4 cm for all long channels. To record extracranial physiological noise, short-distance channels were employed with a source-detector inter-optode distance of about 1 cm [21]. Different cap sizes (54, 56, 58, and 60 cm) were available for various head circumferences, to account for anatomical differences. Time multiplexing was applied to avoid cross-talk between optodes. During measurements, optodes were covered from external light sources by using an opaque cover and all optode cables were securely attached to the participants closing at their back, while leaving enough freedom to not introduce mechanical movement artifacts (Figure 3c; photograph with the opaque cover removed).

LSL. For synchronization purposes of the two independent recording systems (EEG and fNIRS), the Lab Streaming Layer (LSL) was employed. The LSL provided an environment in which synchronized data could be captured from different sources, with synchronized timestamps being provided to the various measurement systems [22]. These timestamps were then incorporated into the measurement data prior to transmission. A custom programmed Raspberry Pi 3 model B (Raspberry Pi Foundation, Cambridge, UK) was utilized to transform the digital photodiode signals (see photodiode) into triggers, which were also transmitted to the LSL. All three data streams within the LSL were then recorded by a single measurement computer using the open-source LSL LabRecorder [23]. The synchronized timestamps guaranteed the synchronization of the data streams.

### 2.3. Participants

A total of four students/faculty members from the Institute of Sport and Exercise Sciences, University of Münster, Germany, participated in this study. Two females and one male (mean age ± SD: 30.3 ± 7.6; 22–37 years) participated in the experiment. Additionally, one 27-year-old male participated in a pre-test for the study. All participants were healthy, had normal or corrected to normal vision, and no history of neurological or psychiatric disorders. Prior to the commencement of the experiment, each participant provided written informed consent. Participation in the experiment constituted a voluntary part of coursework or was voluntary. No compensation was offered to the participants. The study was approved by the local Ethics Committee of the University of Münster, Faculty of Psychology and Sport and Exercise Sciences, Münster (protocol code: 2024-12-RS-Breaking the Freeze–Behavioral and Neural Aspects of Freezing of Gait and Vibrotactile Cueing in Parkinson’s Disease”; date of approval: 4 May 2024).

### 2.4. Task and Procedure of the Piloting

#### 2.4.1. Task and Experimental Setup

Experiment 1—Checkerboard (synchronization between GRAIL and EEG). The checkerboard experiment was designed to measure visually evoked potentials (VEPs) utilizing the EEG in order to verify the synchrony between EEG and GRAIL. A VEP is a known physiological phenomenon that typically can be measured over the occipital region of the brain 100 ms after a visual stimulation (e.g., [24] (pp. 256–259)). The physiological timespans for VEPs were then compared to the VEP timespans measured in our experiment, thereby demonstrating that the synchrony of our systems was sufficient to show these phenomena that occur within the millisecond range.

The primary task of the checkerboard experiment required participants to focus on a fixation point (See Section 2.2. Measurement Systems and Synchronization—The Components of the System–Virtual Scenarios) located at the center of a checkerboard. This was performed in a seated position. The checkerboard’s squares alternated colors at a frequency of 0.5 Hz. The color-changing phase comprised 120 stimulations over a period of four minutes. Prior to and following the visual stimulations, a 35 s period was allotted for participants to remain still in order to allow for a return to baseline brain activation. The experiment concluded with the final resting phase.

Experiment 2—Start-to-Go (synchronization between GRAIL and fNIRS). The experiment was designed to measure event-related cerebral hemodynamic activity associated with movement planning and execution with fNIRS. The canonical hemodynamic response function is typically characterized by an initial decrease in oxygenated hemoglobin (HbO_2_) within 1–2 s after movement onset, reflecting the immediate oxygen consumption by the involved neural networks. This initial decrease is followed by a delayed increase in HbO_2_, peaking at around 5–8 s, due to reactive cerebral blood flow increase. Finally, the HbO_2_ levels gradually return to baseline typically within 15–20 s post-movement as the neuronal activity stabilizes. In this experiment, the event onset per trial was the onset of the walking movements, which was determined through the start of the shift of the center of pressure (COP) on the force plates. A typical cerebral hemodynamic response was expected after movement onset, demonstrating synchronicity between the two measurement systems. Concurrently, the EMG activity of the right M. tibialis anterior was measured in relation to the movement onset. An EMG activity is expected shortly before the movement onset calculated by the force plates, as the movement follows the muscular activation. Showing the dependency between COP and EMG supports the content of our setup.

The primary task of the Start-to-Go experiment involved participants undertaking 50 complete, forward walking cycles on a stationary treadmill. The experiment began with participants standing barefoot in a starting position at the back end of the treadmill. The participants were then asked to wait for a beep sound, which occurred after 15 s of standing. Following this, a directive to remain still for an additional 5–10 s before taking steps forward was given. The final initiation of movement was self-directed by the participants. Participants were instructed not to count the duration of the second waiting period, but rather to trust their intuition. The participants were instructed to commence walking by stepping forward with their right foot, ensuring accurate EMG data capture of the movement onsets. After taking a step with the right and then the left foot, the participants were instructed to place their right foot alongside the left and pause briefly. Subsequently, they were to walk backwards to their original starting position. This sequence was repeated until 50 walking cycles had been completed.

#### 2.4.2. Procedure

**Figure 4:**
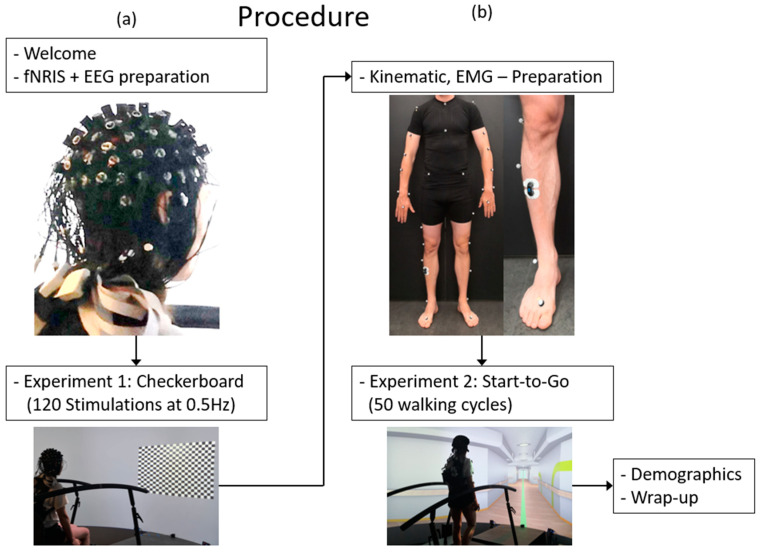
Flow chart of the test procedure. (**a**) The first half of the experiment with preparation of the EEG/fNIRS Setup and the checkerboard task; (**b**) the second half of the experiment with marker and EMG placement, the Start-to-Go task, and the wrap-up.

Each session, which lasted up to three hours, commenced with the arrival of the participants and the signing of consent forms. This initial phase was followed by the measurement of head circumference and body dimensions, which were necessary for the selection of appropriate EEG cap size and setting up the motion capture. Participants were then fitted with an EEG cap equipped with EEG electrodes and fNIRS optodes (Figure 4a, upper insert). These preparatory procedures required approximately two hours before being proceeded by one hour of testing. Following the initial general preparation, the checkerboard experiment (Figure 4a, right insert; see Section 2.4.1. Task and Experimental Setup) was conducted. Participants were provided with detailed instructions and then received the 120 visual stimulations. Following the completion of the first experiment, preparations for the second experiment, Start-to-Go, commenced. 

Participants were equipped with VICON markers according to the Plug-in Gait marker set [17] and EMG electrodes on the right musculus tibialis anterior in accordance to the SENIAM recommendations [18] (Figure 4b, upper insert). A visual inspection of the EMG signal and calibration of the VICON cameras were conducted. Participants were provided with detailed instructions for the experimental procedure of Experiment 2, Start-to-Go (Figure 4b, lower insert; see Section 2.4.1. Task and Experimental Setup), and subsequently proceeded to take the 50 steps required. In order to minimize discomfort experienced by participants as a result of the fNIRS optodes, the duration of the experiment was kept as short as possible. Finally, demographic data, including age and gender, were collected through a questionnaire.

### 2.5. Preprocessing and Data Analysis

#### 2.5.1. Preprocessing and Data Analysis Experiment 1—Checkerboard

EEG. The data of the EEG recording were preprocessed using MNE-Python software Version 1.6.0 [25] with the following packages: Numpy [26], Matplotlib [27], Itertools [28], Pandas [29]. After loading, truncating, and visually inspecting the data, a band-pass filter (0.01–40 Hz) and a Notch filter (50 Hz) were applied. The data were then re-referenced to a common average. An independent Component Analysis (fastICA) was used for the detection and correction of ocular artefacts. The data were then segmented into trials, with a duration from −500 ms before to 400 ms after each stimulus. Event-related potentials were first detrended, followed by baseline correction (−500 ms to −100 ms prior to each stimulus onset) and averaged across trials.

#### 2.5.2. Preprocessing and Data Analysis Experiment 2—Start-to-Go

Kinetic. The analysis of movement onsets via the force plate involves the examination of the COP data in both the anteroposterior (AP) and mediolateral (ML) directions. The force plate data were analyzed using MATLAB R2023a (MathWorks, Natick, MA, USA) with custom scripts. The data were filtered using a zero-phase second-order low-pass Butterworth filter with a cut-off frequency of 5 Hz and a sampling rate of 1000 Hz. The derivatives of the filtered time series for both directions were combined as the square root of the sum of squares. The combined data were analyzed to identify the point at which the first crossing of the threshold of 0.3 (arbitrary unit) occurs. The local minimum nearest to this threshold crossing and not exceeding a maximum height of 0.05 (arbitrary unit) was designated as the movement onset. This procedure for selecting the movement onset was selected following visual inspection of the COP data, as it most accurately represented the cessation of normal swaying while standing and the commencement of the stepping movement. Steps exhibiting unusual CoP patterns around the movement onset or significant changes in the CoP during the baseline were excluded from the analysis after visual inspection. A total of nine steps across one participant were excluded. For the other two participants, no steps were excluded.

EMG. EMG data were processed using MATLAB R2023a (MathWorks, Natick, MA, USA) in accordance with the protocol described elsewhere [30], which included baseline correction, rectification of the EMG data, and the application of a moving average filter across 100 data points. The movement onsets, calculated with the COP, were then annotated within the filtered data. The kinetic and EMG data were then plotted in relation to one another in order to demonstrate the dependency between the two.

fNIRS. The event-related hemodynamic response function related to the above-described step movement onset was extracted from the fNIRS signal. Data were processed using MNE Python [25] with fNIRS-specific processing steps supported by the MNE-NIRS plugin. In addition to the python packages referenced above (cf. EEG preprocessing), the following fNIRS-specific packages were used: Statsmodels (fNIRS) [31], Nilearn (fNIRS) [32]. All raw data were recorded in the universal *.snirf format. First, raw data were imported to MNE python, visually inspected for good signal quality, and truncated to relevant data sections, if necessary. Event annotations were imported based on the above described movement onset detection. Afterwards, all raw data were converted to optical density data, and subsequently into concentration changes in HbO_2_ and deoxygenated hemoglobin (HbR) by employing the modified Beer–Lambert Law [33]. A bandpass filter was applied (low cut-off at 0.02 Hz, high cut-off at 0.2 Hz) to attenuate irrelevant systemic signal frequencies. Systemic fluctuations and mechanical artifacts, typically due to head or larger body movements, were reduced in all long channels by identifying these components in the nearest short channels. Finally, all data were epoched based on individual step events into time sections of 5 s prior to and 15 s after movement onset.

## 3. Results

To test the synchronization within the GRAIL system with the two neurophysiological measurement systems (EEG and fNIRS), we developed two test scenarios designed to elicit typical neurophysiological responses.

### 3.1. Experiment 1—Checkerboard—Synchronization between GRAIL and EEG 

**Figure 5:**
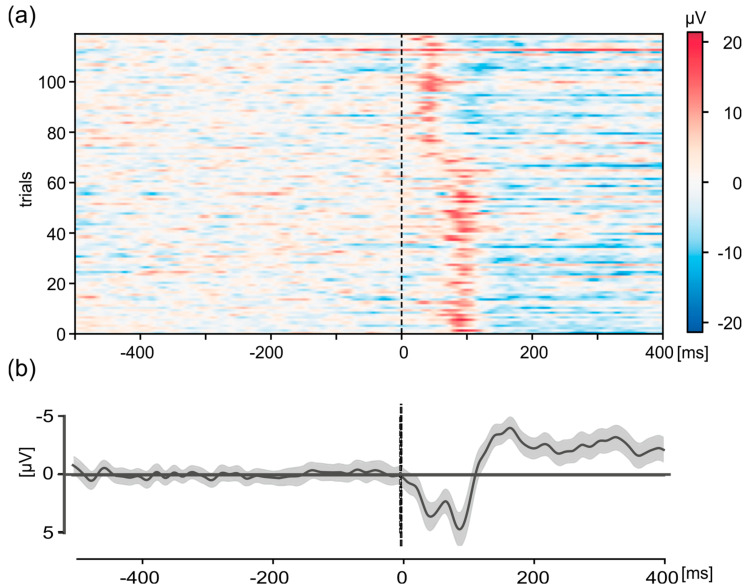
Visual-evoked potentials (VEPs) of a checkerboard pattern of the Oz electrode in the interval 500 ms before stimulus-onset to 400 ms after for the pre-test participant, measured with the old version of the script that resulted in shifts of signal presentation onto the photodiode. These shifts resulted in a shift in the VEP in the analysis, resulting in two peaks at two different time points. Shown are (**a**) representation of the individual trials (*n* = 120). The activity is color-coded, see scale on the right. (**b**) Grand average. Shown are the mean value (thick black line) and the 95% confidence interval (gray shaded area). For (**a**,**b**), data were detrended and baseline corrected (−500 to −100 ms).

The use of VEPs enabled the identification of a discrepancy in the desired synchronization of the system within the GRAIL prior to the commencement of the study. The evaluation of the VEPs from a test measurement revealed a shift in the P100 at the Oz electrode after approximately half the duration of the VEP measurement (Figure 5). Shown are the individual trials (*n* = 120) in the interval from 500 ms before stimulus onset to 400 ms afterwards. Consequently, two peaks were identified in the average graph. One peak with an amplitude of 3.8 µV was found at a latency of 44 ms and another peak with an amplitude of 4.8 µV was found at a latency of 88 ms. This phenomenon cannot be explained by physiological principles and indicates a malfunction in the system.

**Figure 6:**
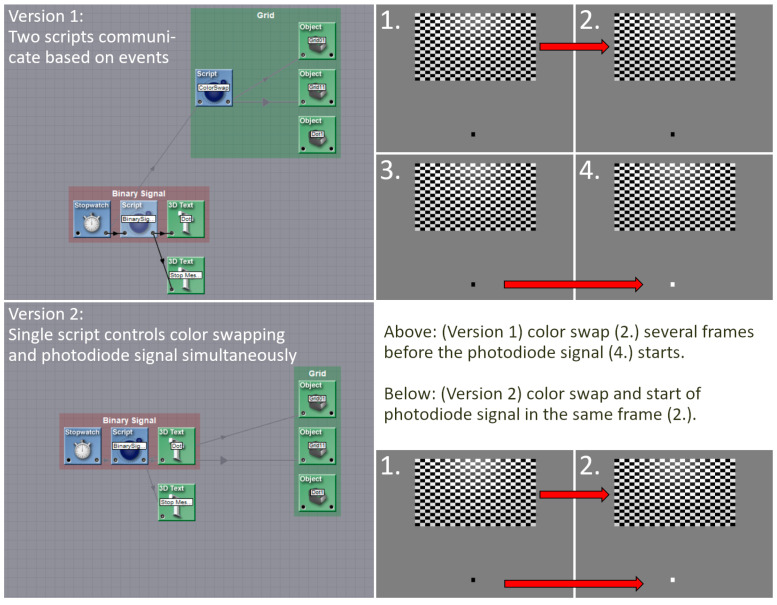
Shown are the old version (upper part, Version 1) of the programming with two scripts and the new programming (lower part, Version 2) with one script. The old version used a script that triggered the checkerboard stimulation and an event which activated the second script. The second script sent a signal onto the photodiode. This method resulted in a jittered delay 12 ms to 56 ms between stimulation and signal, which made an analysis in a millisecond range impossible. The integration of the scripts into a single entity, which initiates both the stimulation and the signal, has resolved this issue.

During the troubleshooting process, the source of the issue that caused the shift in the VEPs was identified. That is, during the test measurements, two scripts were employed to trigger the stimuli in the checkerboard experiment and simultaneously transmit the signals to the photodiode. One script was used to trigger the stimuli, while a second script was used to send the signals to the photodiode. The signal script was initiated via an event from the stimulation script. Given that the scripts were executed at a much higher frequency (300 Hz) than the generation of the frames for the output (60 Hz), and that the output was linked to the creation of a new frame, it would have been expected that both scripts would have been executed within the same frame. However, it can be seen from the VEPs and a subsequent screen capture of the output to the projectors that this was not always the case. The photodiode signal was received in the LSL with a variable delay of approximately 12 ms to 56 ms in relation to the stimuli. However, the integration of the two scripts into a single script resolved this issue and ensured that the signal was transmitted at the same time as the stimuli (Figure 6), thereby ensuring synchronicity within the GRAIL system.

**Figure 7:**
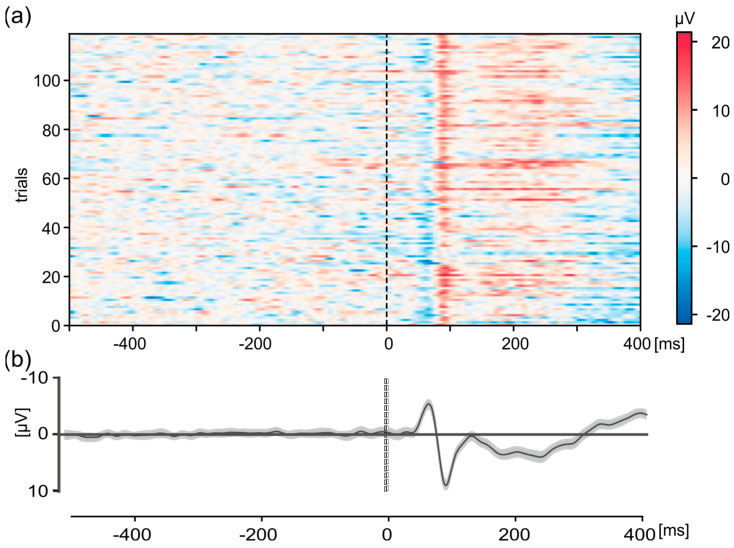
Visual-evoked potentials (VEPs) of a checkerboard pattern of the Oz electrode in the interval 500 ms before stimulus-onset to 400 ms after for one exemplary participant. (**a**) Representation of the individual trials (*n* = 120). The activity is color-coded, see scale on the right. (**b**) Grand average. Shown are the mean value (thick black line) and the 95% confidence interval (gray shaded area). For (**a**,**b**), data were detrended and baseline corrected (−500 to −100 ms).

The improved setup made it possible to measure VEPs in true relation to the presentation of the checkerboard stimulation in the GRAIL system. An example of such a measurement for electrode Oz is shown in Figure 7. As in Figure 5, the individual trials (*n* = 120) for one exemplary participant in the interval from 500 ms before stimulus onset to 400 ms afterwards (Figure 7a) as well as the overall average (Figure 7b) with 95% confidence interval in the same period are shown. The data are baseline-corrected using the mean activity 500 ms before stimulus onset. The differences in neuronal activity from baseline activity are color-coded for the individual trials (Figure 7a). The grand-average shows the typical P100 positivity after stimulus-onset with a latency of ~90 ms and an amplitude of 9.15 µV (Figure 7b). Additionally, a typical negativity, N75, is shown with a latency of 65 ms after the stimulation and a peak amplitude of −5.39 µV. This activity is clearly visible in the individual trials (Figure 7a). This shows that our system is able to measure EEG data with a constant delay of about 10 ms, thereby showing synchronicity both within the GRAIL and between the GRAIL and EEG.

### 3.2. Experiment 2—Start-to-Go—Synchronization between GRAIL and fNIRS

To further support the synchronicity within the GRAIL system, EMG activity is shown in relation to the force plate data, synchronized via the movement onset. To show the synchronous connection and inter-relatability between the fNIRS and the GRAIL, the hemodynamic brain activity during the start-to-go experiment was related to the kinetic movement onset.

**Figure 8:**
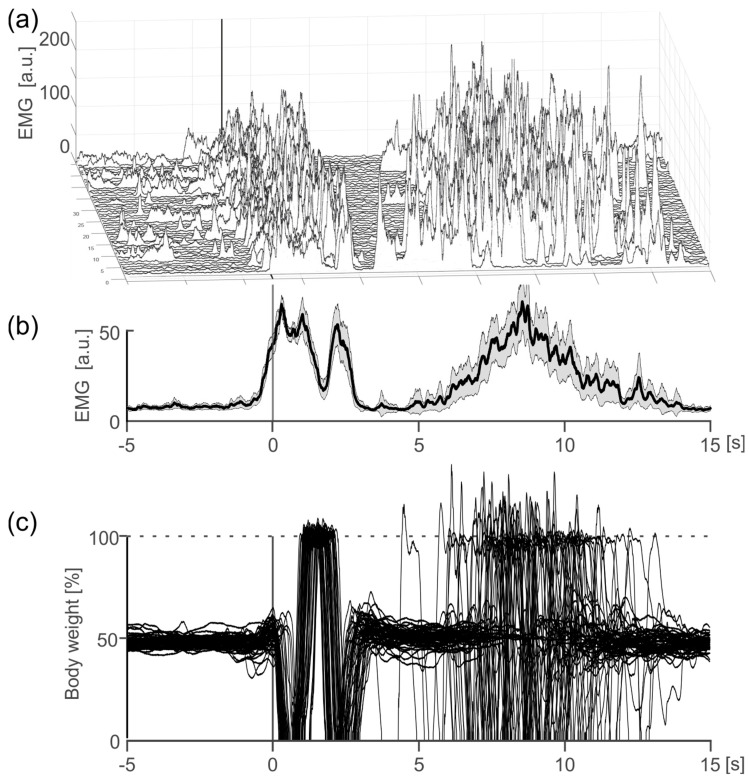
EMG activation of the right musculus tibialis anterior in the interval from 5 s before a voluntary step movement to 15 s after one exemplary participant. (**a**) Illustration of the individual trials (*n* = 50). (**b**) Mean EMG activation. Shown are the mean value (thick black line) and the 95% confidence interval (gray shaded area). (**c**) Overlay of ground reaction forces for the right foot of all trials. The forces are scaled to percentage of bodyweight.

In order to further support the internal validity of our system, we demonstrate that the GRAIL system is capable for synchronous recording with external measurement systems in addition to the internal force plates. For this, the EMG of the tibialis anterior muscle was recorded as an example in addition to the force plate. The graph (Figure 8) shows that the EMG signal accurately shows activation just before the calculated movement onset from the ground reaction force. As the externally measured ground reaction force movement depends on the internal activation of the muscles, a corresponding activation of the tibialis anterior just before the calculated movement onset is physiological. This result shows external measurement systems like the EMG can be recorded in the GRAIL system within VICON with sufficient synchronization.

To show the synchronicity between the GRAIL and the fNIRS, the fNIRS system measured the cerebral hemodynamic response function related to the planning and execution of forward step movements. These changes occur relatively slowly compared to electric neuronal activity. For example, the peak of the hemodynamic response function (in HbO_2_) is typically observed about 5–8 s after movement (or stimulus) onset. A corresponding change in hemodynamic activity in motor and sensorimotor areas can be expected with every voluntary step. Accordingly, the participants were instructed to take a voluntary step forward on the treadmill (see Methods).

**Figure 9:**
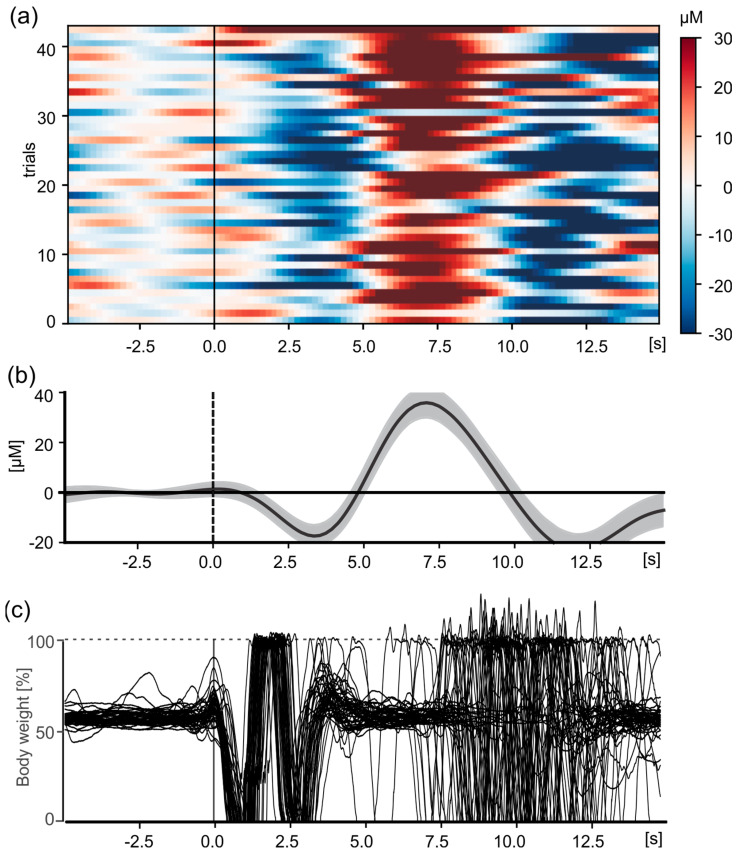
Change in HbO_2_ in the sensorimotor cortex (CCPz) in the interval from 5 s before a voluntary step movement to 15 s after. (**a**) Illustration of the individual trials (*n* = 43) for one exemplary participant. The changes are color coded, see scale on the right. (**b**) Mean hemodynamic response function. Shown are the mean value (thick black line) and the 95% confidence interval (gray shaded area). For (**a**,**b**), the data were baseline corrected using the mean change from 5 to 0.1 s before movement onset. (**c**) Overlay of ground reaction forces for the right foot of all trials. The forces are scaled to percentage of bodyweight.

Hemodynamic changes were measured as a function of the initiation of a voluntary step movement. The onset of the step movement was determined in each trial based on the change in the ground reaction forces of the force plates (GRAIL system). The overlaid force curves are shown in Figure 9c. The average hemodynamic response waveform, which resembles a typical event-related hemodynamic response function, is shown in Figure 9b. After the start of the step, oxygen is consumed, leading to an initial drop in HbO_2_, after which an inflow of oxygen is supplied after about 3 s (Figure 9a), reaching a peak around 7 s after movement onset. After peaking at 7 s, HbO_2_ concentrations decrease with a typical rebound effect observed between 10–15 s (Figure 9b, 8–11 s), before finally returning to baseline level. This typical hemodynamic response can be observed in almost every trial (Figure 9a). In summary, it can be seen that hemodynamic changes from the fNIRS system are measured synchronously with force measurement data from the GRAIL system.

## 4. Discussion

It has been recommended to conduct gait analysis under ecologically valid conditions in order to accurately mirror real-world locomotion [8]. Furthermore, it is essential that neurophysiological activity can be recorded during gait in order to gain a deeper understanding of the neuronal correlates and to understand changes in gait patterns due to age or gait disorders [6]. In order to facilitate valid and reliable evaluations, it is critical that all measurement systems are synchronized without a time delay. We therefore aimed to validate our system, which allows synchronized measurement of neurophysiological activity and movement data under ecologically valid conditions. Using VEPs, event-related changes in HbO_2_, EMG activations, as well as kinetic data from force plates, we were able to show that our system can record movement and neurophysiological data synchronously in real-time, and that these data can be analyzed in relation to each other and in relation to visual stimuli. We developed and implemented technical solutions to make these measurements synchronously. We used a photodiode to compensate for delays within the displaying system, and a Raspberry Pi to feed the photodiode signals into our neurophysiological measurement systems. By additionally recording the photodiode signals within the movement recordings, we were then able to relate the different systems to each other. By showing the dependency between EMG and force plate data, we further supported the internal validity of the GRAIL system. Through the internal synchronicity of the VICON measurement system, kinematic data can also be captured synchronously, even though this has not been shown in this proof-of-principle study.

This proof-of-principle study demonstrates that our system integration achieved sufficient real-time synchronization by incorporating a photodiode and a Raspberry Pi into the GRAIL system. It further indicates that the way the scripts are integrated might induce additional time delays, if conducted incorrectly. Our configuration allows for the synchronous analysis of previously independent neurophysiological and kinetic measurement systems. The results might be also adapted for other systems as the GRAIL, as all systems used in this setup to achieve the synchronicity are independent of the GRAIL system and could be used in systems other than the GRAIL. By use of such a system integration, it allows for the measurement of inter-individual and intra-individual differences in physiological signals, as we were able to replicate measurements of known phenomenon with latencies in the millisecond range. Utilizing the GRAIL system, which incorporates virtual reality, our approach supports realistic and effective gait analysis investigations.

The measurement setup for EEG and fNIRs used in this study (see Figure 3b) was relatively heavy, with the main weight being carried by means of a backpack. This ensured that the weight did not represent a significant burden on the participants. In addition, the setup did not restrict movement and allowed for natural and unrestricted activity. The cables were fixed to the body in such a way that unrestricted head movement was guaranteed. This also allowed unrestricted rotation of the head. This was confirmed a posteriori by the absence of any complaints from the participants about the strain or restricted mobility. Based on these observations, it can be assumed that patient groups will also be able to bear the weight. This setup allows for the performance of ecologically valid gait analyses with different participants, including those of varying age and health status. It can be used with healthy younger and older individuals, as well as those with sensorimotor disorders such as stroke, Parkinson’s, or Cerebellum (e.g., [34,35,36]).

### Limitations

One limitation of our system arises from the programming of our virtual scenarios. As we had to adapt our programming in the run-up to the study by combining different scripts, we lost the modularity computation that would otherwise have been present in our system. Consequently, any change to the system necessitates adaptation of all virtual scenarios, rather than just one standard signal transmission script. For instance, should the coding of the photodiode signals be revised, all scenarios that utilize the photodiode must be adapted due to the absence of modularity. This necessitates additional work and increases the susceptibility to errors. It appears that this limitation is an inherent consequence of the current configuration, as the modularity employed at the outset has proven to be the source of difficulty with synchronization.

Another limitation is the GRAIL’s level of virtual reality. On the classification system proposed by Slosar et al. [37], the GRAIL can be categorized as a personal computer (PC), as the projection screen does not fully surround the user. This also has some advantages, as it enables researchers to monitor participants during an experiment without the use of cameras and from close proximity. This allows researchers to aid participants if they need to, which might be necessary when researching patient groups. The utilization of projectors also permitted the integration of a photodiode, which would be impractical with a head-mounted display (HMD). The additional weight that would be carried by participants when utilizing an HMD could also present challenges when conducting research with patient groups.

## 5. Conclusions

In order to relate kinematic events to neurophysiological activity, it is crucial to record data synchronously. The methods employed in our experimental setup, namely a photodiode, a custom programmed Raspberry Pi, and the LSL, enable synchronization of a complex system such as the GRAIL with independent measurement systems for the measurement of neurophysiological activity. Such a system allows for the measurement of valid neurophysiological phenomena in relation to kinetic and kinematic measures.

## Data Availability

The data and code that support the findings of this study are available upon reasonable request from the corresponding author, D.F.K.
